# Health Characteristics of Adults Unable to Complete Medicaid Renewal During the Unwinding Period

**DOI:** 10.1001/jamahealthforum.2025.0092

**Published:** 2025-03-21

**Authors:** Aparna Soni, Justin Blackburn

**Affiliations:** 1 Richard M. Fairbanks School of Public Health, Indiana University Indianapolis, Indianapolis

## Abstract

**Importance:**

Medicaid redetermination affects millions of people annually in the US, but little is known about beneficiaries who lose coverage during the process. In early 2023, after the COVID-19 pandemic mandate to provide continuous coverage was lifted, states resumed redetermination of eligibility and disenrollment of individuals who were no longer eligible. Medicaid disenrollment rose rapidly during this unwinding period, yet little is known about the health and financial characteristics of individuals who were unable to complete the Medicaid renewal process.

**Objective:**

To assess levels of self-reported mental health, functional health, and financial security among current and former Medicaid enrollees during the Medicaid unwinding period.

**Design, Setting, and Participants:**

This repeated cross-sectional study used nationally representative survey data from multiple waves of the US Census Bureau Household Pulse Survey to assess the health and financial characteristics of 131 384 current and former working-age (19-64 years) Medicaid enrollees interviewed between January 2023 and September 2024. Estimated linear regression models were used to compare measures among current Medicaid enrollees, former enrollees who could not complete renewal (procedural disenrollees), and former enrollees who dropped Medicaid for other reasons (nonprocedural disenrollees). Analysis was performed in December 2024.

**Exposure:**

Medicaid enrollment status and likely reason for disenrolling (procedural vs nonprocedural).

**Results:**

The total sample comprised 131 384 current and former working-age Medicaid enrollees (mean [SD] age, 41.9 [12.5] years; 82 378 females [62.7%]; 22 467 Black [17.1%], 32 715 Hispanic/Latino [24.9%], 62 276 White [47.4%], and 13 927 individuals of other races or multiracial [10.6%]), more than half of whom were parents and of lower socioeconomic status. Compared with current Medicaid enrollees, procedural disenrollees were 3.3 percentage points (pp) more likely to report anxiety (95% CI, 1.6 to 4.9 pp); frequent worrying, 3.3 (95% CI, 1.8 to 4.8) pp; little interest in things, 2.4 (95% CI, 1.0 to 3.8) pp; depression, 2.5 (95% CI, 1.1 to 3.9) pp; food insecurity, 3.6 (95% CI, 2.6 to 4.7) pp; difficulty seeing, 2.0 (95% CI, 1.0 to 3.0) pp; difficulty hearing, 1.1 (95% CI, 0.4 to 1.8) pp; and difficulty remembering things, 1.4 (95% CI, 0.1 to 2.7) pp; however, they were 1.3 pp less likely to report difficulty with mobility (95% CI, −2.5 to −0.2 pp). There was no statistically significant difference between groups in difficulty with bathing and dressing or difficulty understanding things.

**Conclusions and Relevance:**

This cross-sectional study found that adults who could not complete the Medicaid renewal process and were procedurally disenrolled during the unwinding period had higher mental health needs, worse functional health, and lower financial security than current enrollees and nonprocedural disenrollees. These findings raise concerns regarding the potential consequences of administrative barriers and Medicaid coverage disruptions on vulnerable populations.

## Key Points

**Question:**

What are the mental health, functional health, and financial characteristics of adults who could not complete the Medicaid renewal process (ie, procedural disenrollees) during the early unwinding period?

**Findings:**

This cross-sectional study found that compared with current Medicaid enrollees, those unable to complete the renewal process were statistically significantly more likely to report anxiety, frequent worrying, little interest in things, depression, food insecurity, and difficulty seeing, hearing, and remembering.

**Meaning:**

These findings raise concerns regarding the potential consequences of administrative barriers and Medicaid coverage disruptions for people in vulnerable conditions.

## Teaser

This cross-sectional study assesses levels of self-reported mental health, functional health, and financial security among current and former Medicaid beneficiaries during the Medicaid unwinding period.

## Introduction

Medicaid beneficiaries must routinely redemonstrate their eligibility for Medicaid each year through a renewal, redetermination, or recertification process. Its purpose is to ensure that those who are no longer eligible for Medicaid do not continue to receive its benefits. However, beneficiaries who are still eligible may inadvertently lose their coverage if they do not complete the required renewal process.

During the COVID-19 public health emergency (PHE), the Medicaid redetermination process was temporarily paused. The Families First Coronavirus Response Act of 2020 included a continuous coverage requirement that incentivized states to keep Medicaid enrollees continuously enrolled without eligibility redeterminations through the end of the PHE period in exchange for enhanced federal funding.

In late 2022, the US Congress passed the Consolidated Appropriations Act, which included legislation to end the continuous coverage provision. In April 2023, states resumed requiring redetermination of Medicaid eligibility and disenrolling individuals no longer eligible. Medicaid disenrollment rose rapidly during this “unwinding period.” Approximately 31% of nearly 81 million Medicaid enrollees disenrolled by September 2024, nearly 70% due to procedural reasons, which included the inability or failure to complete paperwork.^1^ Because no eligibility determination was made for these individuals, it is unknown how many of the 25 million beneficiaries who were disenrolled were truly no longer eligible for Medicaid compared with how many lost benefits despite remaining eligible.

There is widespread concern that this massive rise in procedural disenrollment will disproportionately affect people with health conditions who need care. With the conclusion of the Medicaid unwinding, millions of enrollees will resume annual redetermination of eligibility. Substantial literature^2,3,4^ that predates this unwinding shows that low-income individuals who lost Medicaid due to changes in eligibility requirements experienced reduced access to care and lower financial security. However, these findings may or may not apply to those who lost coverage due to procedural reasons during the unwinding. The unique policy context of this Medicaid unwinding period provides an opportunity to understand this population and how administrative processes may differentially affect fair access to coverage.

Existing studies^1,5,6^ largely rely on administrative data to measure the number of beneficiaries who lost Medicaid coverage during the Medicaid unwinding. Although administrative data provide reliable estimates of the number and some basic demographic characteristics of procedural disenrollees, these data are not suited for understanding the health and financial needs of individuals who could not complete Medicaid renewal. To our knowledge, there is currently only 1 empirical study that rigorously examined the health characteristics of those who lost Medicaid coverage due to renewal requirements during the unwinding. McIntyre et al^7^ conducted a survey of low-income households in 4 southern US states in 2023 and found that 6 months into the unwinding, adults who exited Medicaid were more likely to be insured yet also reported greater challenges to accessing care. This study provides important insights into access to care and health care utilization among Medicaid disenrollees during the unwinding period, but the study sample is limited to 4 southern states. Regional studies may not be reflective of the whole country. Thus, there is a need for national-level estimates of the health and socioeconomic characteristics of former Medicaid enrollees who were procedurally disenrolled during the unwinding period.

This study sought to fill that gap by using the US Census Bureau Household Pulse Survey (HPS), a nationally representative survey designed to rapidly capture and publish data on household experiences during and after the COVID-19 pandemic. The HPS identifies individuals who tried to continue their Medicaid coverage but were unable to complete the renewal process, which we defined as likely “procedural disenrollment.” The HPS allows identification of this population, who presumably wanted coverage and believed themselves to be eligible, a group distinct from those who disenrolled themselves due to ineligibility.

Our study builds on the findings of McIntyre et al^7^ by leveraging national survey data to study a sample of recent Medicaid enrollees from all 50 states. We also build on previous work by Rumalla et al^8^ who used the HPS data to highlight racial and ethnic disparities among those who lost Medicaid coverage for procedural reasons during the unwinding period. Using methods similar to those of Rumalla et al, our objective was to provide timely insights into the mental health, functional health, and financial security characteristics of working-age adults who were disenrolled from Medicaid due to procedural reasons compared with current enrollees and nonprocedural disenrollees.

## Methods

This cross-sectional study was exempted by the institutional review board of Indiana University because it used only publicly available deidentified data; informed consent was also waived for this reason. We followed the Strengthening the Reporting of Observational Studies in Epidemiology (STROBE) reporting guideline.

### Study Data and Population

We analyzed 20 waves of the HPS, a nationally representative survey of adults conducted from January 4, 2023, through September 16, 2024. eTable 1 in Supplement 1 lists interview dates for each wave. The survey was not conducted in November and December 2023, and data on Medicaid disenrollment were unavailable for January to March 2024; therefore, these time frames were excluded from our analysis. The HPS collects biweekly data from approximately 68 460 respondents per wave; demographic information, including race and ethnicity, are self-reported.

We restricted the study sample to current and former adult Medicaid enrollees, aged 19 to 64 years, who had Medicaid coverage at any point in the 1 to 2 years before their interview. We excluded adults older than 65 years because they were eligible for Medicare throughout our study period. Our study sample included 131 384 individuals across 20 waves. The total weighted number of respondents was approximately 33 million per wave.

**Figure 1.**
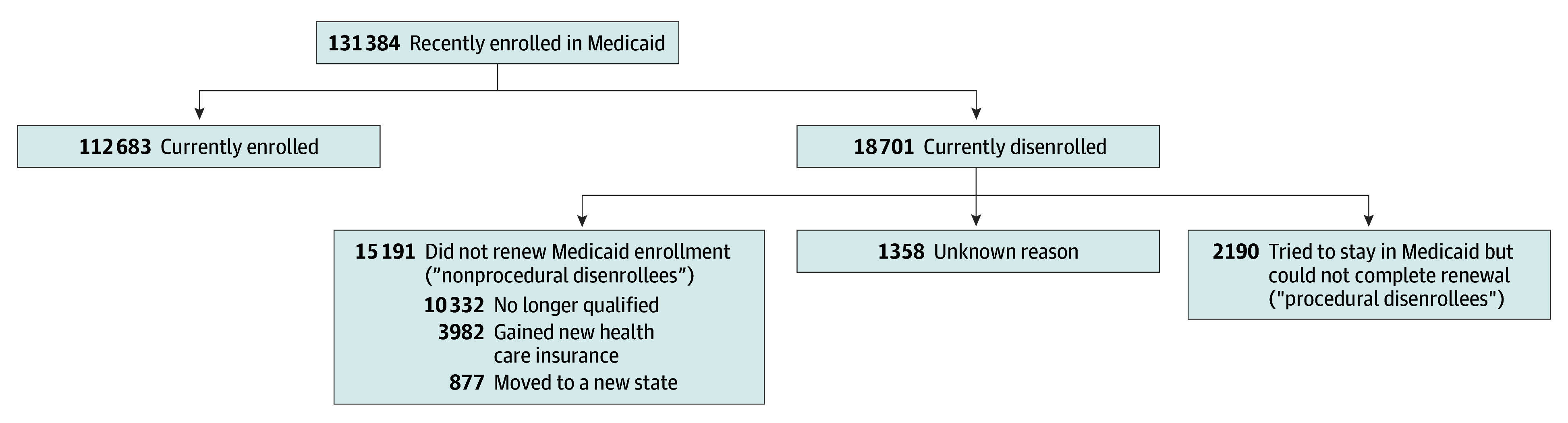
Study Sample of Recent Medicaid Enrollees (N = 131 384) From the Household Pulse Survey (HPS) Calculations based on HPS week 53 through cycle 9 (January 2023-September 2024). Percentages are weighted by HPS sampling weights.

HPS participants reported their current insurance status, whether they were enrolled in Medicaid at any point in the 1- to 2-year period preceding the interview, and why they disenrolled from Medicaid. Those interviewed before August 2023 were asked if they had Medicaid coverage at any point since January 2022, and those interviewed after August 2023 were asked if they had Medicaid coverage at any point since January 2023. In the sensitivity analyses, we determined that the change in look-back period did not substantively affect our results. We categorized respondents into 3 groups: those who continued enrollment in Medicaid, we termed *currently enrolled* (n = 112 683); tried to stay in Medicaid but could not complete the renewal process, we termed *likely procedural disenrollment* (n = 2190); and formerly enrolled in Medicaid but lost coverage for another reason (listed in Figure 1), we termed *nonprocedural disenrollment* (n = 16 511).

The HPS categorization of former Medicaid enrollees does not map precisely onto the conception of procedural and nonprocedural disenrollment, as reported by US states. We believe that the response “tried to stay in Medicaid but could not complete the renewal process” is as close as we can get to identifying those who lost Medicaid due to procedural disenrollment. Notably, since no eligibility determination could be made, these former enrollees were not outright denied coverage. Rather, their enrollment expired because states lacked information regarding the enrollee’s intention for coverage. This same classification scheme was used by Rumalla et al^8^ to identify procedural disenrollees. Because our definition of procedural disenrollment was not a perfect measure, our analysis did not accurately measure the number of procedural disenrollees nationwide. However, the advantage of the HPS is that it provides a rich set of sociodemographic, health, and financial characteristics of this at-risk group of former Medicaid enrollees. Although administrative data can better capture the number of procedural disenrollees during unwinding, our study provides important data on the characteristics of these individuals.

### Statistical Analysis

For the main analysis, we estimated linear probability regression models in which the key independent variables were indicators for whether the respondent was a current Medicaid enrollee (reference category), tried to stay in Medicaid but could not complete the renewal process (likely procedural disenrollee), or lost Medicaid for another reason (nonprocedural disenrollee). We controlled for respondents’ self-reported age, sex, race and ethnicity (using categories provided by HPS), educational attainment, marital status, parental status, household size, household income (as a percentage of the federal poverty level), employment status, and state of residence through a vector of state-fixed effects. We weighted all estimates using the HPS person-level sampling weights.

We evaluated 3 categories of outcomes: mental health—whether the respondent reported high levels of anxiety, frequent worrying, little interest in things, and high levels of depression nearly every day over the past 2 weeks^9^; functional health—whether the respondent reported a lot of difficulty seeing, hearing, remembering things, walking around or climbing stairs, bathing or dressing, and understanding things; and financial security—whether the respondent reported having health insurance coverage and often not having enough to eat. eTable 2 in Supplement 1 provides detailed definitions for these outcome variables.

Analyses were conducted in Stata, release 18 (StataCorp LLC), in July to December 2024. Statistical tests were 2-tailed and *P* < .05 was considered statistically significant, and *P* < .10, marginally statistically significant.

## Results

**Figure 2.**
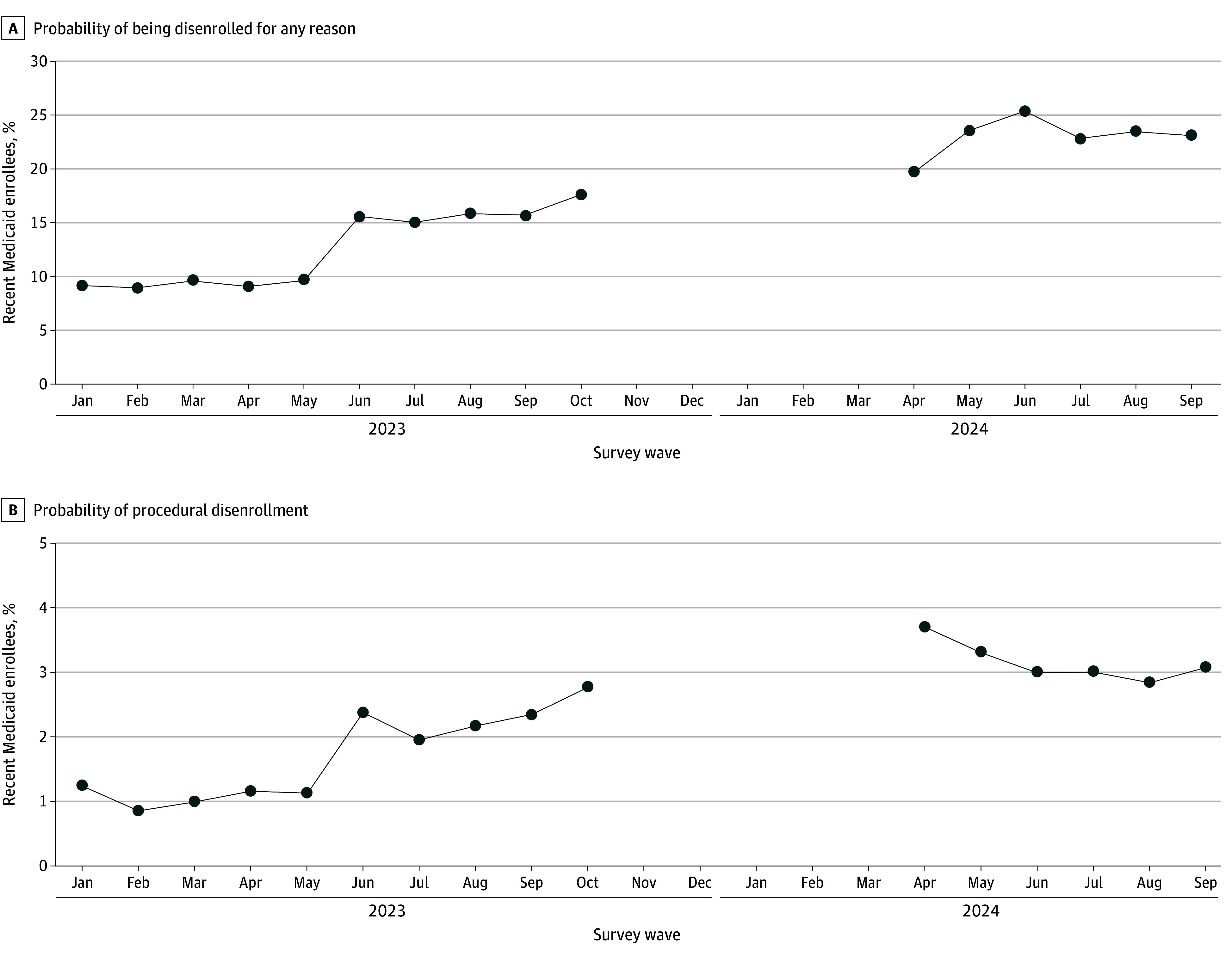
Trends in Medicaid Disenrollment Among 131 384 Current and Former Enrollees Over Time Calculations based on Household Pulse Survey week 53 through cycle 9 (January 2023-September 2024, except for October 2023 through February 2024). Estimates are weighted by Household Pulse Survey sampling weights.

The total study population included 131 384 current and former working-age Medicaid enrollees (mean [SD] age, 41.9 [12.5] years; 82 378 females [62.7%] and 49 006 males [37.3%]; 22 467 Black [17.1%], 32 715 Hispanic/Latino [24.9%], 62 276 White [47.4%], and 13 927 individuals of other races or multiracial [10.6%]), more than half of whom were parents and of lower socioeconomic status. Figure 2 presents trends in Medicaid disenrollment. Among those who were recently enrolled in Medicaid, the probability of disenrollment increased from 9% in January 2023 to 23% by September 2024 (Figure 2A). Likely procedural disenrollment increased rapidly during this period (Figure 2B), from 1.2% in January 2023 to 3.7% by April 2024 and 3.1% by September 2024 (Figure 2B). eFigure 1 in Supplement 1 presents trends over time in nonprocedural reasons for disenrolling from Medicaid. Although we present these trends to highlight changes in Medicaid disenrollment among our study sample over time, we caution that these self-reported data should not be interpreted as national estimates of procedural disenrollment in Medicaid. The HPS data underestimate the incidence of procedural disenrollment.

Among former Medicaid enrollees surveyed in the HPS, approximately half (52.6%) reported that they no longer qualified (eFigure 2 in Supplement 1). Another 20.7% chose to drop Medicaid coverage. A smaller proportion, 13.5%, reported that they tried to keep Medicaid but were not able to complete the renewal process (likely procedural disenrollment). There was substantial geographic variation in procedural disenrollment, with rates higher than 20% in Alaska, Arkansas, Washington DC, Hawaii, Indiana, and Montana (eFigure 3 in Supplement 1).

Table 1 presents demographic characteristics of the 3 categories of respondents. Compared to current Medicaid enrollees, procedural disenrollees were younger, less educated, and more likely to be single, parents, male, employed, and Black or Hispanic. Our regression analysis controlled for these demographic characteristics.

**Table 1.** Demographic Characteristics of Study Population, by Medicaid Enrollment Status^a^

Characteristic	No. (%)			
	Total study population	Medicaid enrollment status		
		Current enrollees	Procedural disenrollees	Nonprocedural disenrollees
Participants, No.	131 384	112 683	2190	16 511
Age, mean (SD), y	41.9 (12.5)	42.3 (12.5)^b^	39.8 (12.1)^b^^,^^c^	39.3 (12.0)^c^
Female	82 314 (62.7)	71 779 (63.7)^b^	1236 (56.4)^c^	9339 (56.6)^c^
Male	49 070 (37.4)	40 904 (36.3)^b^	954 (43.6)^c^	7172 (43.4)^c^
Race and ethnicity				
Black	22 433 (17.1)	19 060 (16.9)^b^	446 (20.4)^b^^,^^c^	2912 (17.6)^c^
Hispanic	32 651 (24.9)	27 491 (24.4)^b^	683 (31.2)^b^^,^^c^	4446 (27.0)^c^
White	62 314 (47.4)	54 115 (48.0)^b^	823 (37.6)^b^^,^^c^	7423 (45.0)^c^
Other^d^	13 986 (10.6)	12 017 (10.7)	238 (10.9)	1731 (10.5)
Educational attainment				
<High school	17 753 (13.5)	15 609 (13.9)^b^	308 (14.1)^b^	1839 (11.1)^c^
High school	50 654 (38.6)	43 833 (38.9)^b^	958 (43.8)^b^^,^^c^	5848 (35.4)^c^
Some college	43 599 (33.2)	37 611 (33.3)^b^	662 (30.2)^b^^,^^c^	5340 (32.3)^c^
≥College	19 378 (14.7)	15 631 (13.9)^b^	261 (11.9)^b^^,^^c^	3485 (21.1)^c^
Married	49 716 (38.0)	41 785 (37.2)^b^	769 (35.3)^b^^,^^c^	7161 (43.5)^c^
Parent	71 094 (54.1)	61 384 (54.4)^b^	1231 (56.2)^b^	8476 (51.3)^c^
Household size, mean (SD)	3.9 (2.0)	3.9 (2.0)^b^	3.9 (2.0)^b^	3.8 (1.9)^c^
% of Federal poverty level, mean (SD)	194.6 (164.5)	185.4 (153.4)^b^	189.1 (169.2)^b^	259.8 (216.2)^c^
Employed	67 967 (52.1)	55 532 (50.0)^b^	1173 (54.2)^b^^,^^c^	11 218 (68.4)^c^

^a^ Calculations based on Household Pulse Survey week 53 through cycle 9 (January 2023-September 2024). Estimates are weighted by Household Pulse Survey sampling weights.

^b^ Significantly different from nonprocedural disenrollees (*P* < .05).

^c^ Significantly different from current enrollees (*P* < .05).

^d^ Includes individuals who self-reported race and ethnicity as Asian, any other race, or multiracial.

Next, we evaluated the association between procedural disenrollment and health and financial measures. eFigure 4 in Supplement 1 presents unadjusted means of our outcome variables for each category of respondents. For nearly all measures, procedural disenrollees were worse off than both current Medicaid enrollees and nonprocedural disenrollees. For example, 14% of current Medicaid enrollees reported high depression levels compared with 17% of procedural disenrollees and 12% of nonprocedural disenrollees.

Table 2 presents regression-adjusted estimates of the association of procedural disenrollment with health and financial measures. These data show that compared to current Medicaid enrollees, procedural disenrollees were 3.3 percentage points (pp) (16%) more likely to report high anxiety (95% CI, 1.6-4.9 pp), 3.3 pp (19%) more likely to report frequent worrying (95% CI, 1.8-4.8 pp), 2.4 pp (18%) more likely to report little interest in things (95% CI, 1.0-3.8 pp), and 2.5 pp (18%) more likely to report high depression (95% CI, 1.1-3.9 pp). For all these measures, procedural disenrollees were also statistically significantly worse than nonprocedural disenrollees. However, nonprocedural disenrollees were less likely to report adverse mental health conditions than current enrollees.

**Table 2.** Regression Analysis for Association of Medicaid Disenrollment With Health and Financial Measures Among 131 384 Current and Former Enrollees^a^

Enrollee status	Estimate (95% CI)											
	Mental health				Functional health						Financial security	
	High anxiety	Frequent worrying	Little interest in things	High depression	Difficulty seeing	Difficulty hearing	Difficulty remembering	Difficulty walking/ climbing stairs	Difficulty bathing/ dressing	Difficulty understanding	Any insurance	Often not enough to eat
Study population, No.	124 086	123 951	123 963	123 970	124 098	123 848	124 034	124 038	124 067	124 112	123 930	123 966
Procedural disenrollees	3.25^b^^,^^c^ (1.63 to 4.88)	3.30^b^^,^^c^ (1.77 to 4.83)	2.39^b^^,^^c^ (1.01 to 3.77)	2.48^b^^,^^c^ (1.08 to 3.88)	2.02^b^^,^^c^ (1.04 to 3.00)	1.11^b^^,^^c^ (0.43 to 1.78)	1.39^c^^,^^d^ (0.13 to 2.65)	−1.31^d^ (−2.46 to −0.16)	0.51^c^ (−0.19 to 1.20)	−0.34 (−0.91 to 0.23)	−53.52^b^^,^^c^ (−54.20 to −52.83)	3.64^b^^,^^c^ (2.60 to 4.67)
Nonprocedural disenrollees	−1.00^b^ (−1.69 to −0.31)	−1.18^b^ (−1.83 to −0.53)	−0.75^d^ (−1.34 to −0.17)	−0.65^d^ (−1.24 to −0.06)	0.54^d^ (0.12 to 0.96)	−0.37^d^ (−0.65 to −0.08)	−1.10^b^ (−1.64 to −0.57)	−2.60^b^ (−3.09 to −2.11)	−0.60^b^ (−0.90 to −0.31)	−0.31^d^ (−0.55 to −0.06)	−26.10^b^ (−26.39 to −25.81)	0.09 (−0.35 to 0.52)
Current enrollees, mean (%)	20.5	17.3	13.5	14.0	6.2	2.9	11.0	10.4	3.1	2.1	100.0	7.1

^a^ Calculations were based on the Household Pulse Survey, week 53 through cycle 9 (January 2023 to September 2024). The study sample was restricted to recent adult Medicaid enrollees (N = 131 384). The Reference group was current Medicaid enrollees (n = 112 683). We report regression-adjusted percentage point differences between those who disenrolled because they could not complete the renewal process (ie, procedural disenrollees), those who disenrolled for some other reason (ie, nonprocedural disenrollees), and current Medicaid enrollees (reference category). The analyses controlled for respondents’ age, sex, race and ethnicity, educational attainment, marital status, parental status, household size, household income, employment status, and state of residence. For comparison, the last row presents the (nonregression adjusted) mean for current Medicaid enrollees. Estimates are weighted by Household Pulse Survey sampling weights.

^b^ *P *< .01.

^c^ Procedural disenrollees group is statistically significantly different from the nonprocedural disenrollees group with *P *< .01.

^d^ *P *< .05.

 Next, we examined functional health outcomes. Compared to current enrollees, procedural disenrollees were 2.0 pp (12%) more likely to report difficulty seeing (95% CI, 1.0 to 3.0 pp), 1.1 pp (38%) more likely to report difficulty hearing (95% CI, 0.4 to 1.8 pp), 1.4 pp (13%) more likely to report difficulty remembering (95% CI, 0.1 to 2.7 pp), and 1.3 pp (13%) less likely to report difficulty walking or climbing stairs (95% CI, −2.5 to −0.2 pp). There was no detectable association between disenrollment and difficulty bathing and dressing or difficulty understanding.

Table 2 also shows that procedural disenrollees had worse measures of financial security than both current enrollees and those who disenrolled for other reasons. Procedural disenrollees were 53.5% less likely to be insured than current enrollees (95% CI, −54.2 to −52.8), vs a 26.1% smaller likelihood of coverage for nonprocedural disenrollees (95% CI, −26.4 to −25.8 pp). Compared to current enrollees, procedural disenrollees were 3.6 pp (51%) more likely to face food insecurity (95% CI, 2.6 to 4.7 pp).

eTable 3 in Supplement 1 presents heterogeneity tests in which we estimated regressions separately by race and ethnicity, sex, parental status, and age group. We used postestimation seemingly unrelated estimation commands to formally test whether coefficient sizes were statistically significantly different across demographic subgroups. For mental health measures, we found the strongest associations between Medicaid disenrollment and mental health for non-Hispanic White and non-Hispanic Black respondents. For most other subgroups, coefficient sizes were not statistically significantly different across demographic subgroups. For functional health measures, the differences between procedural disenrollees and current enrollees were largest for non-Hispanic White adults and older adults aged 50 to 64 years. The disparity in food insecurity between procedural disenrollees and current enrollees was similar across demographic subgroups.

eTable 4 in Supplement 1 shows that our results are robust to numerous sensitivity analyses, including using alternate definitions of mental health measures; controlling for respondents’ week of interview; dropping state fixed effects; and restricting our study window to either before or after the HPS lookback period changed in August 2023.

Finally, eFigure 5 in Supplement 1 presents a scatterplot comparing state-level rates of likely procedural disenrollment in the HPS to rates published by the Kaiser Family Foundation.^1^ We found a high correlation between the 2 measures, which provides additional confidence in our HPS measure of procedural disenrollment.

## Discussion

Using repeated cross-sectional waves of the nationally representative HPS survey during the Medicaid unwinding in 2023 and 2024, we found that likely procedural disenrollment from Medicaid was associated with lower levels of self-reported mental health, functional health, and financial security. Those unable to complete the renewal process reported higher levels of anxiety, depression, frequent worrying, little interest in things, food insecurity, and difficulty seeing, hearing, and remembering things compared with those who remained continuously enrolled in Medicaid or disenrolled for other reasons. The results of this analysis combined with previously observed racial and ethnic disparities procedural disenrollment^8^ raise concerns about potential consequences of administrative barriers and Medicaid coverage disruptions on populations living in vulnerable situations.

Administrative procedures are required for social safety-net programs to ensure beneficiaries’ eligibility to receive benefits. However, prior literature^10,11^ has demonstrated that administrative burden reduces take-up of Medicaid and other social safety-net programs, particularly among racial and ethnic minority groups^8,12^ and those with limited English proficiency.^13^ Our analysis provides evidence that administrative burdens in Medicaid redetermination may also exacerbate disparities by health and financial status. During the PHE, many enrollees qualified for Medicaid for the first time and may never have had to complete the redetermination process before. Furthermore, outdated contact information may have prevented enrollees from receiving information about the requirements for redetermination, including due dates. Due to the nature of the HPS data, we could not ascertain the specific reasons for inability to complete the renewal process or how many participants were able to subsequently qualify and enroll in coverage.

We observed that those who could not complete renewal had more mental and functional health needs. This appears counter to evidence^6^ prior to the PHE that found that those with greater health care utilization were more likely to renew coverage. Our findings raise concerns because loss of Medicaid coverage has been shown to persistently disrupt health care access and utilization, especially among those with mental health disorders.^3,14^ Our finding that procedural disenrollees were more likely to be food-insecure is also concerning because food insecurity is highest among those with chronic health conditions and associated with greater health care utilization and expenditures.^15,16,17^

Earlier research shows that in other contexts, human-centered outreach efforts (eg, Medicaid navigators) were able to assist beneficiaries with navigating the administrative processes and minimize unnecessary loss of benefits. Additional research is needed to understand the role of state, federal, and community partnership outreach in mitigating adverse effects of the current Medicaid unwinding on medically and financially at-risk populations.

Our observation that those unable to complete the Medicaid renewal process had a multitude of vulnerabilities, including health status, functional limitations, food insecurity, and financial well-being compared to those who either remained continuously enrolled or disenrolled for other reasons raises concerns about equitable access to health coverage. These consequences may be most dire for those who need health care access the most because they have health-related social needs. From a public health perspective, these vulnerabilities may place low-income adults at greater risk of exacerbations of chronic disease, poorer health status, and financial hardships due to poor access to health care.^18,19,20^

Although applications to Medicaid for eligibility determination and continued coverage may be necessary, mitigating the administrative barriers through patient navigators and outreach efforts at the community, state, and federal levels may be important for beneficiaries with the greatest needs. Prior studies^21,22,23^ have shown the benefits of these programs when the Affordable Care Act expanded Medicaid eligibility in most states. These activities are particularly important during times of major or rapid changes, including implementation of new eligibility requirements in Medicaid.^20,24^

### Limitations

The HPS survey data inform us of whether a former Medicaid enrollee reported trying to keep Medicaid but was unable to complete the renewal process. This is an imperfect definition of procedural disenrollment, and our analysis underestimates the true number of procedural disenrollees as captured by administrative data. Relatedly, to the extent that some people who were continuously covered during the PHE were unaware that they were still covered by Medicaid, the denominator of our study (which includes recent Medicaid enrollees, based on self-reports) may have been underestimated. This was a descriptive analysis, and therefore, we cannot ascertain whether procedural enrollment worsened health measures or whether those who were already in worse health were less likely to be able to complete the renewal process. Moreover, the HPS survey data are self-reported and may not capture true reasons for Medicaid coverage loss. We did not control for differences in health outcomes and unwinding processes across states, which may partially explain some of the observed disparity, although including state fixed effects partially addressed this concern. We could not include beneficiaries younger than 19 years because the HPS interviews are only administered to adults. Lastly, the HPS is a repeated cross-sectional survey and cannot be used to determine longitudinal enrollment.

## Conclusions

This cross-sectional study found that adults who could not complete the Medicaid renewal process and were procedurally disenrolled during the unwinding period had higher mental health needs, worse functional health, and lower financial security than current enrollees and nonprocedural disenrollees. These findings raise concerns about potential consequences of administrative barriers and Medicaid coverage disruptions on people living in vulnerable situations. The broad changes to Medicaid policy prompted by the PHE were unprecedented but may provide insights into future policy opportunities for states to refine administrative requirements for enrollment.

## Notes

Supplement 1. eTable 1. Household Pulse Survey Dates of Interview eTable 2. Definitions for Household Pulse Survey Dependent and Independent Variables of Interest eFigure 1. Percent of Recent Medicaid Enrollees that Disenrolled in Medicaid, by Reason for Disenrollment eFigure 2. Reason for Medicaid Disenrollment for Former Medicaid Enrollees eFigure 3. Percent of Recent Medicaid Enrollees that Could Not Complete Renewal Process, by State eFigure 4. Means of Outcome Variables by Medicaid Enrollment Status (Not Regression Adjusted) eTable 3. Heterogeneity Tests eTable 4. Sensitivity Analyses eFigure 5. Scatterplot Showing the Correlation Between Household Pulse Survey’s and Kaiser Family Foundation’s Measures of “Procedural Disenrollment”

Supplement 2. Data Sharing Statement
