# Supplementary material for: Health Characteristics of Adults Unable to Complete Medicaid Renewal During the Unwinding Period
Source: JAMA Health Forum. 2025 Mar 21;6(3):e250092. doi: 10.1001/jamahealthforum.2025.0092 (PMC11929023; doi:10.1001/jamahealthforum.2025.0092)
Supplement: Supplement 1. — eTable 1. Household Pulse Survey Dates of Interview eTable 2. Definitions for Household Pulse Survey Dependent and Independent Variables of Interest eFigure 1. Percent of Recent Medicaid Enrollees that Disenrolled in Medicaid, by Reason for Disenrollment eFigure 2. Reason for Medicaid Disenrollment for Former Medicaid Enrollees eFigure 3. Percent of Recent Medicaid Enrollees that Could Not Complete Renewal Process, by State eFigure 4. Means of Outcome Variables by Medicaid Enrollment Status (Not Regression Adjusted) eTable 3. Heterogeneity Tests eTable 4. Sensitivity Analyses eFigure 5. Scatterplot Showing the Correlation Between Household Pulse Survey’s and Kaiser Family Foundation’s Measures of “Procedural Disenrollment” [file jamahealthforum-e250092-s001.pdf]

Supplemental Online Content

Soni A, Blackburn J. Health Characteristics of Adults Unable to Complete Medicaid Renewal During the Unwinding Period. *JAMA Health Forum.* Published online March 21, 2025.
doi:10.1001/jamahealthforum.2025.0092

eTable 1. Household Pulse Survey Dates of Interview

eTable 2. Definitions for Household Pulse Survey Dependent and Independent Variables of Interest

eFigure 1. Percent of Recent Medicaid Enrollees that Disenrolled in Medicaid, by Reason for Disenrollment

eFigure 2. Reason for Medicaid Disenrollment for Former Medicaid Enrollees

eFigure 3. Percent of Recent Medicaid Enrollees that Could Not Complete Renewal Process, by State

eFigure 4. Means of Outcome Variables by Medicaid Enrollment Status (Not Regression Adjusted)

eTable 3. Heterogeneity Tests

eTable 4. Sensitivity Analyses

eFigure 5. Scatterplot Showing the Correlation Between Household Pulse Survey's and Kaiser Family Foundation's Measures of "Procedural Disenrollment"

This supplemental material has been provided by the authors to give readers additional information about their work.

© 2025 Soni A et al. *JAMA Health Forum*.

eTable 1. Household Pulse Survey Dates of Interview

Survey Wave	Dates of Interview
Week 53	January 4, 2023 to January 16, 2023
Week 54	February 1, 2023, to February 13, 2023
Week 55	March 1, 2023 to March 13, 2023
Week 56	March 29, 2023 to April 10, 2023
Week 57	April 26, 2023 to May 8, 2023
Week 58	June 7, 2023 to June 19, 2023
Week 59	June 28, 2023 to July 10, 2023
Week 60	July 26, 2023 to August 7, 2023
Week 61	August 23, 2023 to September 4, 2023
Week 62	September 20, 2023 to October 2, 2023
Week 63	October 18, 2023 to October 30, 2023
Cycle 1	January 9, 2024 to February 5, 2024
Cycle 2	February 6, 2024 to March 4, 2024
Cycle 3	March 5, 2024 to April 1, 2024
Cycle 4	April 2, 2024 to April 29, 2024
Cycle 5	April 30, 2024 to May 27, 2024
Cycle 6	May 28, 2024 to June 24, 2024
Cycle 7	June 25, 2024 to July 22, 2024
Cycle 8	July 23, 2024 to August 19, 2024
Cycle 9	August 20, 2024 to September 16, 2024

Notes: The Census Bureau's Household Pulse Survey public use files are available at https://www.census.gov/programs-surveys/household-pulse-survey/data/datasets.2023.html#list-tab-1264157801

© 2025 Soni A et al. *JAMA Health Forum*.

eTable 2. Definitions for Household Pulse Survey Dependent and Independent Variables of Interest

HPS Variable Name	Question	Response Choices	Variable Name and Coding Scheme for This Study
Current and Former Medicaid Enrollees			
MEDICAID	Since January 1, 2022, have you ever had Medicaid coverage?<br><br>(In Week 61 and later, this question changed to: Since January 1, 2023, have you ever had Medicaid coverage.)	1) Yes, I had Medicaid coverage but I no longer have it.<br>2) Yes, I currently have Medicaid coverage.<br>3) No, I have not had Medicaid since January 1, 2022.<br>-99) Question seen but category not selected<br><.m>) Missing / Did not report<br><br>(In Week 61 and later, Option 3 changed to, “No, I have not had Medicaid since January 1, 2023.”)	**Enrollment Status**<br>Those who responded 2 to “MEDICAID” were categorized as “Currently Enrolled.” Those who responded 1 to “MEDICAID” *and* 4 to “MEDICAID_NO” were categorized as “Procedural disenrollment.” Those who responded 1 to “MEDICAID” and either 1, 2, 3, or –99, or missing to “MEDICIAD_NO” were categorized as “Non-procedural disenrollment.” Those who responded 3, -99, or missing to “MEDICAID” were not included in analysis.
MEDICAID_NO	What was the main reason you no longer have Medicaid?	1) I gained new coverage and chose to drop Medicaid<br>2) I moved to a new state<br>3) I no longer qualify for Medicaid<br>4) I tried to stay in Medicaid, but I could not complete the renewal process<br>-99) Question seen but category not selected<br><.m>) Missing / Did not report	
Mental Health			
ANXIOUS	Over the last 2 weeks, how often have you been bothered by feeling nervous, anxious, or on edge? Select only one answer.	1) Not at all<br>2) Several days<br>3) More than half the days<br>4) Nearly every day<br>-99) Question seen but category not selected<br><.m>) Missing / Did not report	**High Anxiety**<br><br>Responses 1, 2, and 3 were coded as 0. Response 4 was coded as 1. Responses –99 and missing were not included in analysis.
WORRY	Over the last 2 weeks, how often have you been bothered by the not being able to stop or control worrying? Select only one answer.	(Same as ANXIOUS)	**Frequent Worrying**<br><br>(Same as High Anxiety)

© 2025 Soni A et al. *JAMA Health Forum.*

INTEREST	Over the last 2 weeks, how often have you been bothered by having little interest or pleasure in doing things? Select only one answer.	(Same as ANXIOUS)	**Little Interest in Things**<br><br>(Same as High Anxiety)
DOWN	Over the last 2 weeks, how often have you been bothered by feeling down, depressed, or hopeless? Select only one answer.	(Same as ANXIOUS)	**Depression**<br><br>(Same as High Anxiety)
Functional Health			
SEEING	Do you have difficulty seeing, even when wearing glasses? Select one.	1) No - no difficulty<br>2) Yes - some difficulty<br>3) Yes - a lot of difficulty<br>4) Cannot do at all<br>-99) Question seen but category not selected<br><.m>) Missing / Did not report	**Difficulty Seeing**<br><br>Responses 1 and 2 were coded as 0. Responses 3 and 4 were coded as 1. Responses –99 and missing were not included in analysis.
HEARING	Do you have difficulty hearing, even when using a hearing aid? Select one.	(Same as SEEING)	**Difficulty Hearing**<br><br>(Same as Difficulty Seeing)
REMEMBERING	Do you have difficulty remembering or concentrating? Select one.	(Same as SEEING)	**Difficulty Remembering**<br><br>(Same as Difficulty Seeing)
MOBILITY	Do you have difficulty walking or climbing stairs? Select one.	(Same as SEEING)	**Difficulty Walking/Climbing Stairs**<br><br>(Same as Difficulty Seeing)
SELFCARE	Do you have difficulty with self-care, such as washing all over or dressing? Select one.	(Same as SEEING)	**Difficulty Bathing/Dressing**<br><br>(Same as Difficulty Seeing)

© 2025 Soni A et al. *JAMA Health Forum*.

UNDERSTAND	Using your usual language, do you have difficulty communicating, for example understanding or being understood? Select one.	(Same as SEEING)	**Difficulty Understanding**<br><br>(Same as Difficulty Seeing)
Financial Security			
HLTHINS1 – HLTHINS8	Are you currently covered by any of the following types of health insurance or health coverage plans? Mark Yes or No for each.	Insurance through a current or former employer or union (through yourself or another family member)<br><br>Insurance purchased directly from an insurance company, including marketplace coverage (through yourself or another family member)<br><br>Medicare, for people 65 and older, or people with certain disabilities<br><br>Medicaid, Medical Assistance, or any kind of government-assistance plan for those with low incomes or a disability<br><br>TRICARE or other military health care<br><br>VA (including those who have ever used or enrolled for VA health care)<br><br>Indian Health Service<br><br>Other	**Any Insurance**<br><br>Those who responded yes to being covered by *any* of the health insurance types were coded as 1. Those who responded no to *all* of the insurance types were coded as 0. Those who responded missing to *all* of the insurance types were not included in analysis.
CURFOODSUF	Getting enough food can also be a problem for some people. In the last 7 days, which of these statements best describes the food eaten in your household? Select only one answer.	1) Enough of the kinds of food (I/we) wanted to eat<br>2) Enough, but not always the kinds of food (I/we) wanted to eat<br>3) Sometimes not enough to eat<br>4) Often not enough to eat<br>-99) Question seen but category not selected<br><.m>) Missing / Did not report	**Often Not Enough to Eat**<br><br>Responses 1, 2, and 3 were coded as 0. Response 4 was coded as 1. Responses –99 and missing were not included in analysis.

Notes: The Census Bureau's Household Pulse Survey public use files are available at https://www.census.gov/programs-surveys/household-pulse-survey/data/datasets.2023.html#list-tab-1264157801

© 2025 Soni A et al. *JAMA Health Forum.*

eFigure 1. Percent of Recent Medicaid Enrollees that Disenrolled in Medicaid, by Reason for Disenrollment

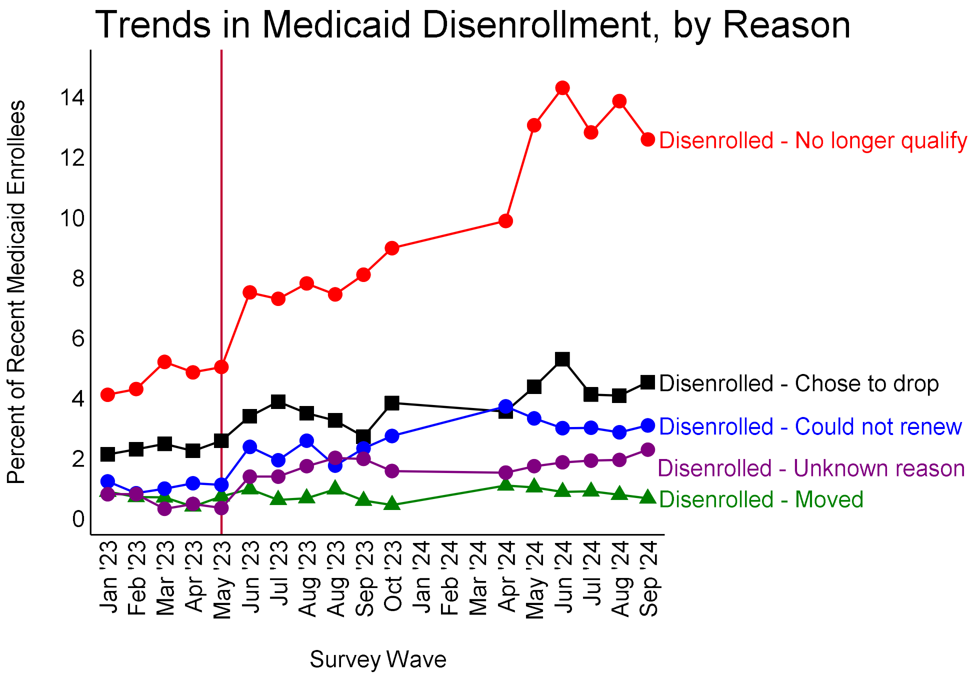

Notes: Authors' calculations based on Household Pulse Survey, Week 53 through Cycle 9 (which covers January 2023 to September 2024). We restricted the study sample to recent adult Medicaid enrollees (N=131,384). Estimates are weighted by HPS sampling weights.

© 2025 Soni A et al. *JAMA Health Forum*.

eFigure 2. Reason for Medicaid Disenrollment for Former Medicaid Enrollees

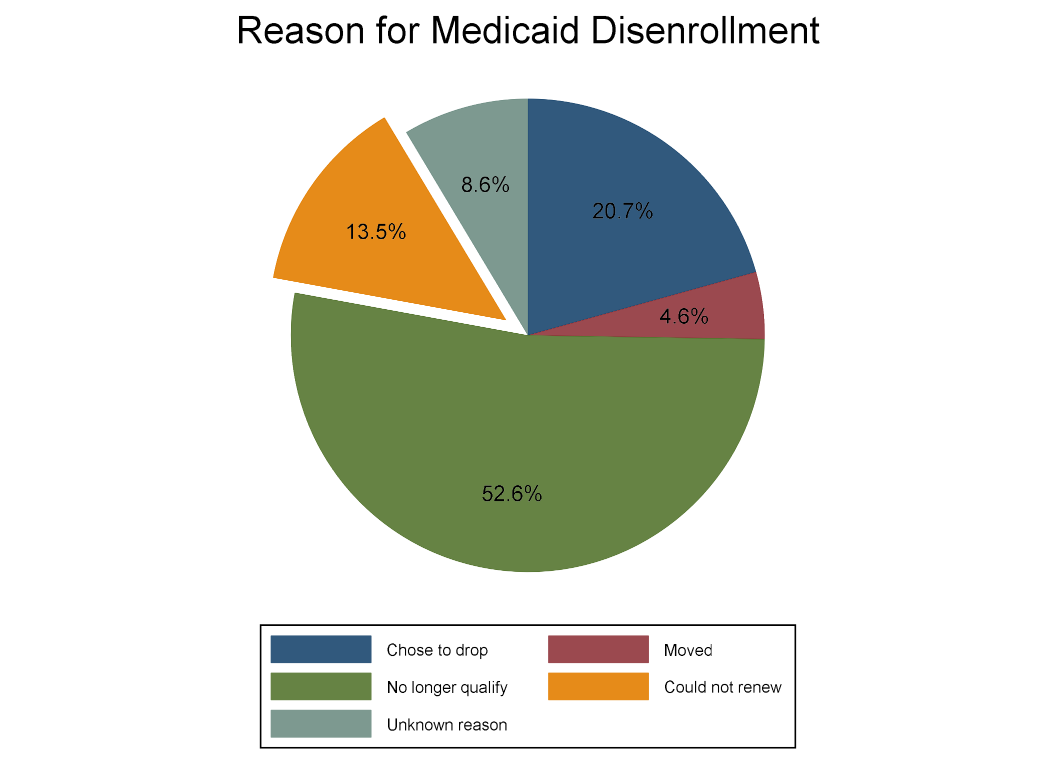

Notes: Authors' calculations based on Household Pulse Survey, Week 53 through Cycle 9 (which covers January 2023 to September 2024). For this figure, sample is restricted to those who recently had Medicaid coverage but are currently disenrolled (N=18,701).

© 2025 Soni A et al. *JAMA Health Forum*.

eFigure 3. Percent of Recent Medicaid Enrollees that Could Not Complete Renewal Process, by State

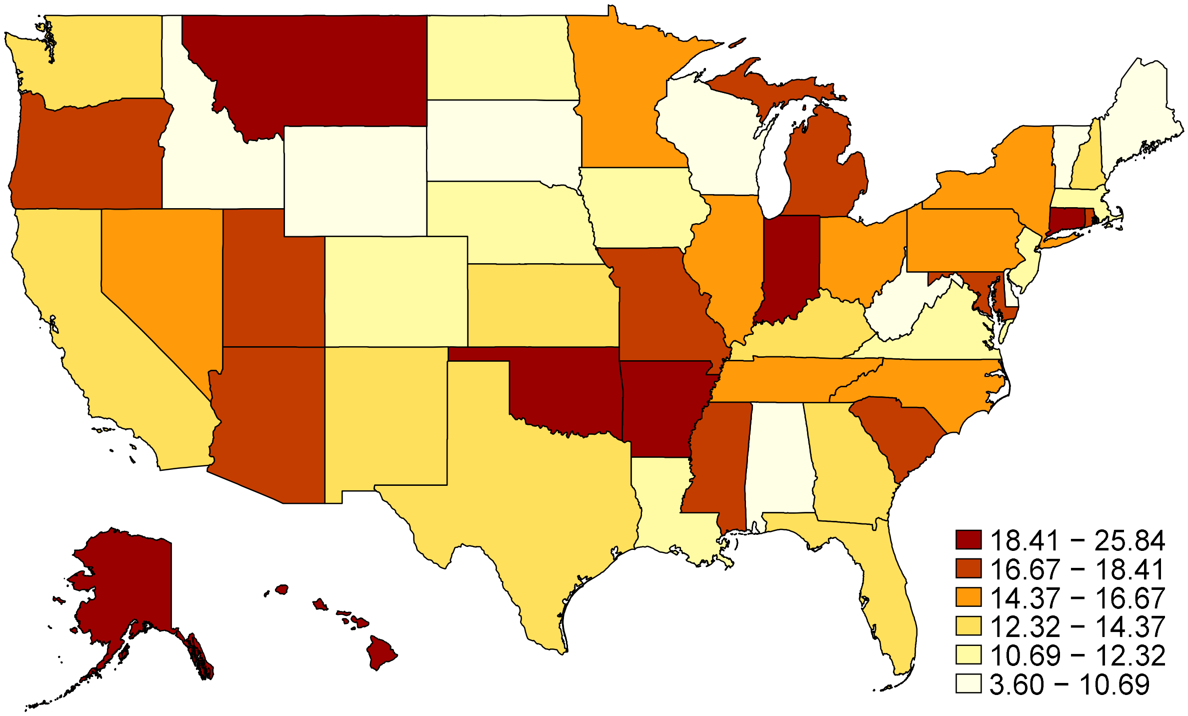

Notes: Authors' calculations based on Household Pulse Survey, Week 53 through Cycle 9 (which covers January 2023 to September 2024). We restricted the study sample to recent adult Medicaid enrollees (N=131,384). Estimates are weighted by HPS sampling weights.

© 2025 Soni A et al. *JAMA Health Forum*.

eFigure 4. Means of Outcome Variables by Medicaid Enrollment Status (Not Regression Adjusted)

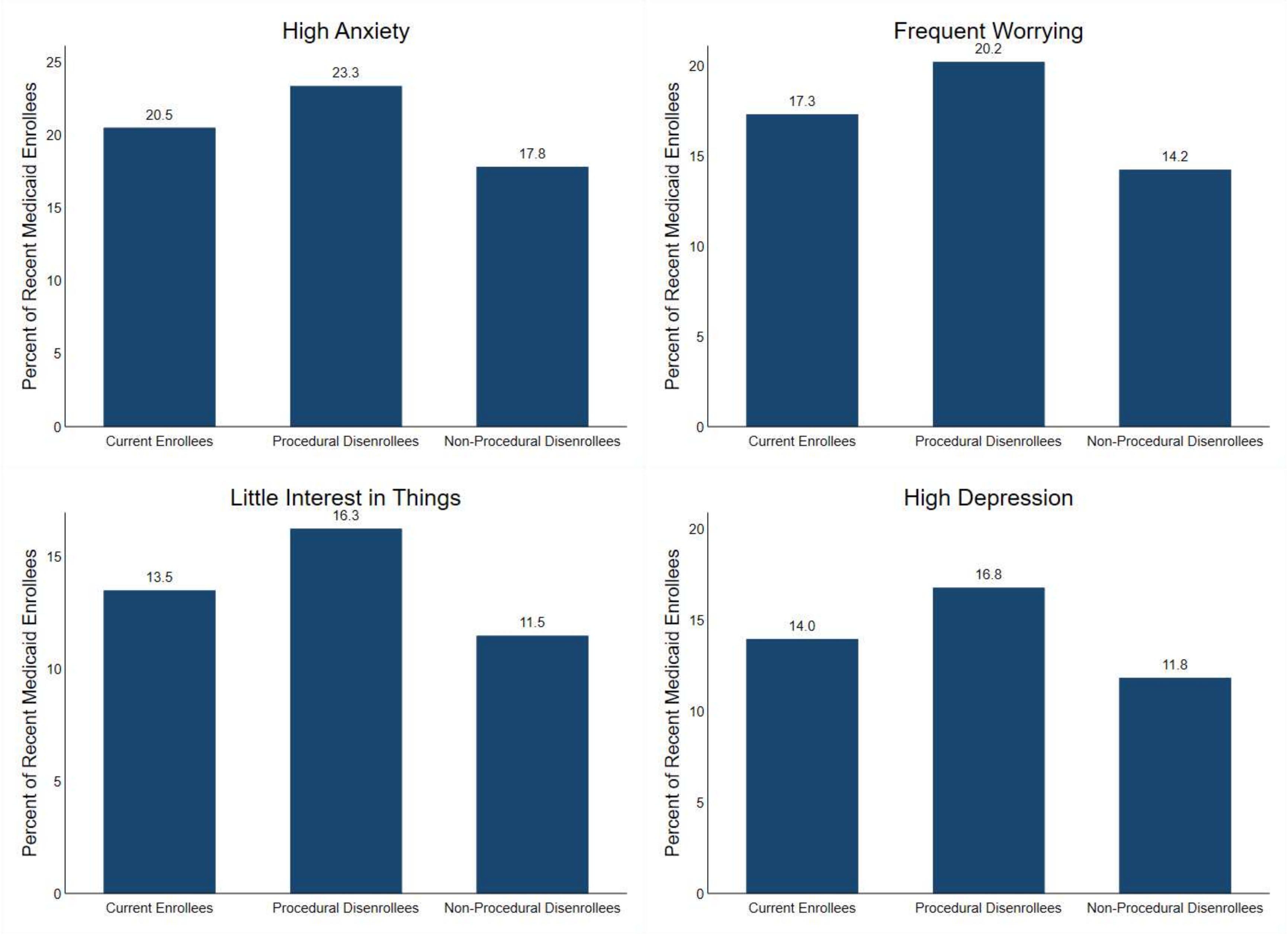

© 2025 Soni A et al. *JAMA Health Forum*.

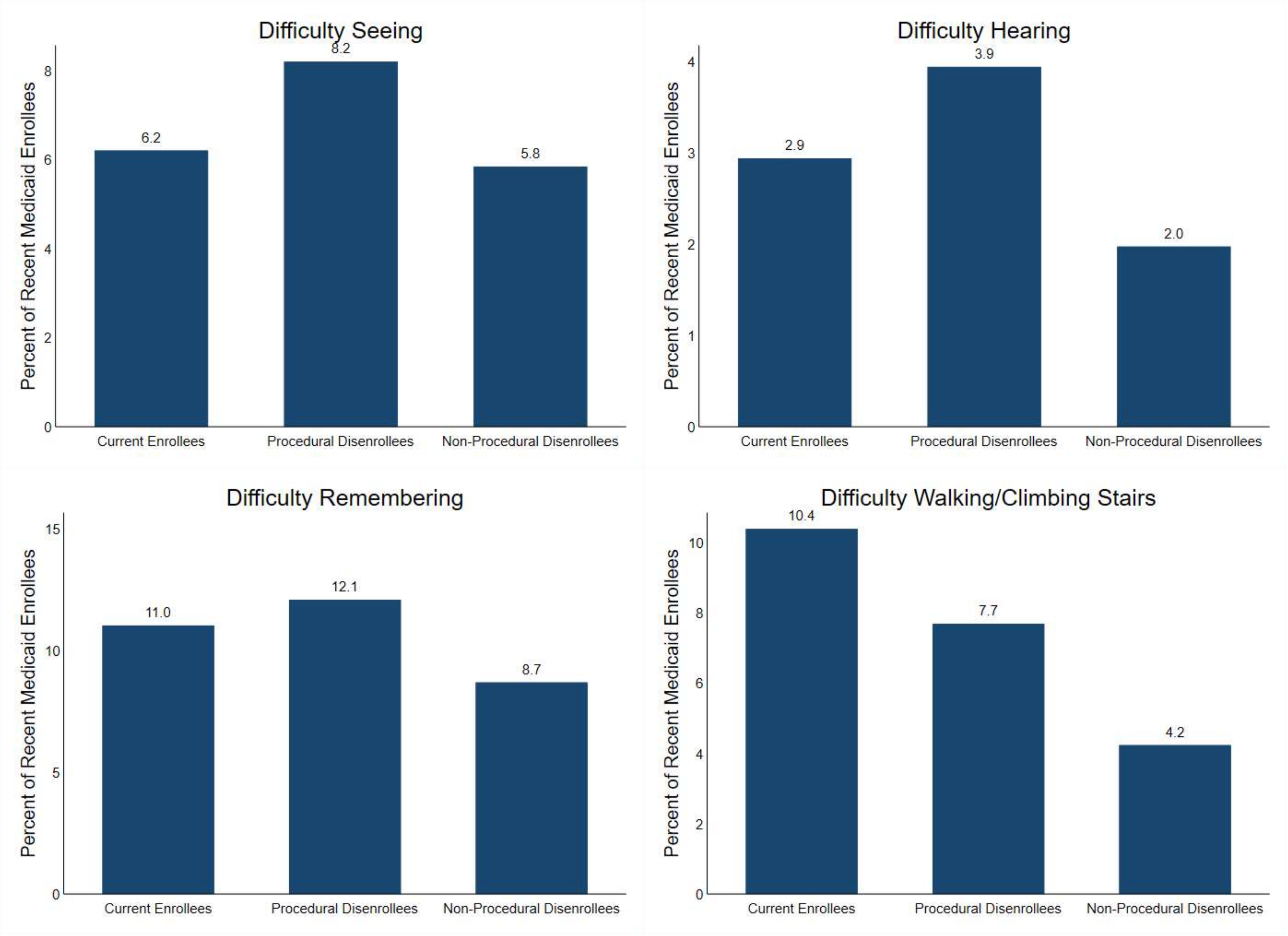

© 2025 Soni A et al. *JAMA Health Forum*.

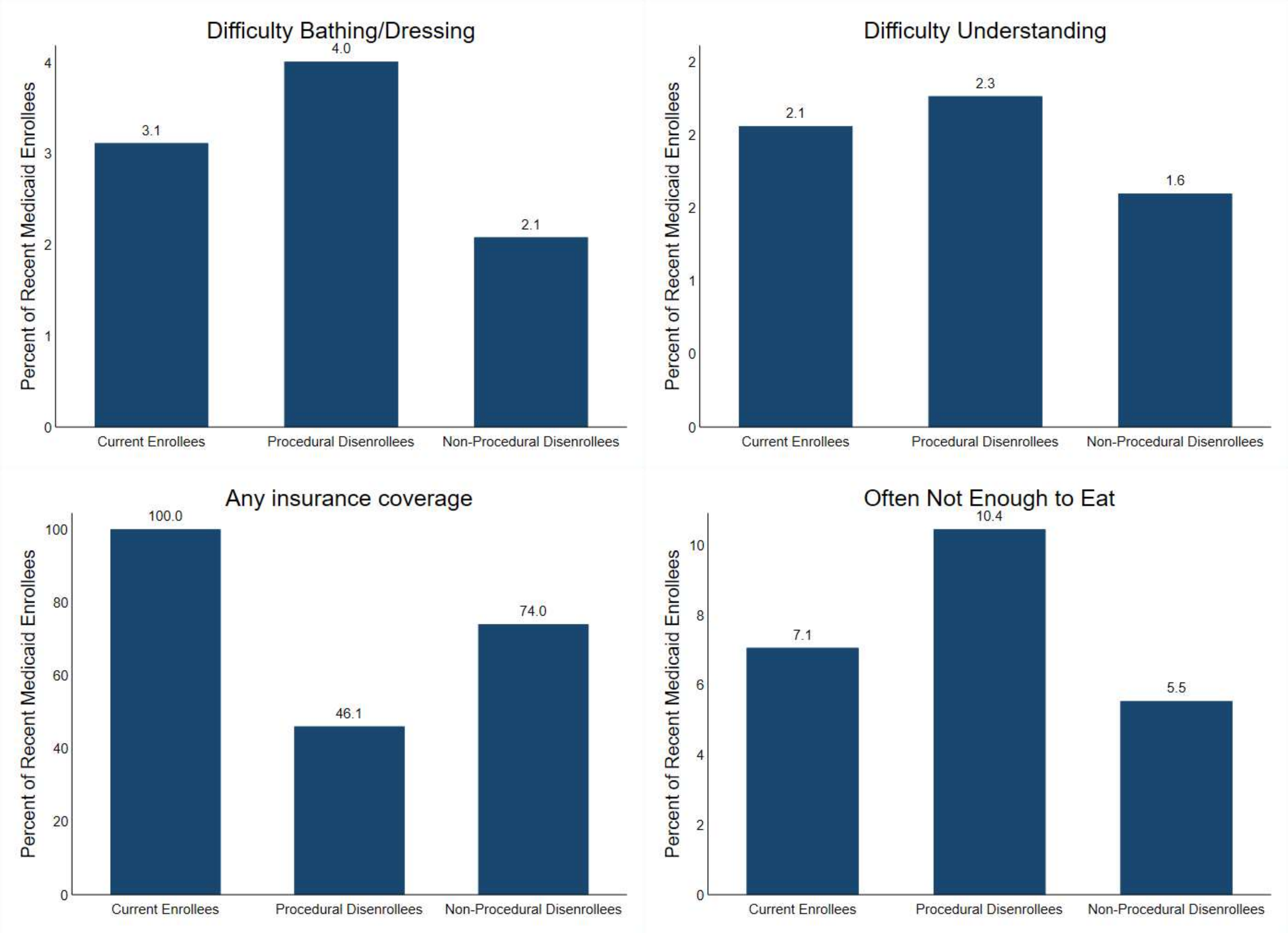

Notes: Authors' calculations based on Household Pulse Survey, Week 53 through Cycle 9 (which covers January 2023 to September 2024). We restricted the study sample to recent adult Medicaid enrollees (N=131,384). Estimates are weighted by HPS sampling weights.

© 2025 Soni A et al. *JAMA Health Forum*.

eTable 3. Heterogeneity Tests

Panel A. Mental Health

	High Anxiety	Frequent Worrying	Little Interest in Things	High Depression
By Race and Ethnicity				
Black, non-Hispanic				
Procedural Disenrollees	-1.64^{b} [-5.46,2.19]	0.11 [-3.64,3.86]	4.93*** [1.50,8.35]	5.17*** [1.74,8.61]
Non-Procedural Disenrollees	-1.29 [-2.99,0.42]	-2.56*** [-4.22,-0.89]	-1.56** [-3.09,-0.04]	-1.26 [-2.80,0.27]
Hispanic				
Procedural Disenrollees	0.36 [-3.30,4.02]	0.65 [-2.80,4.10]	-1.26 [-4.31,1.79]	-2.08 [-5.18,1.02]
Non-Procedural Disenrollees	-2.84*** [-4.52,-1.17]	-3.56^{***,c} [-5.15,-1.98]	-2.50*** [-3.90,-1.10]	-3.23^{***,b} [-4.65,-1.81]
Other race, non-Hispanic				
Procedural Disenrollees	-2.24^{b} [-7.12,2.64]	-3.12^{b} [-7.85,1.61]	1.19 [-3.10,5.48]	1.05 [-3.24,5.35]
Non-Procedural Disenrollees	-2.02* [-4.13,0.09]	-2.71*** [-4.75,-0.67]	-1.60* [-3.46,0.25]	-2.26** [-4.12,-0.40]
White, non-Hispanic (Comparison Subgroup)				
Procedural Disenrollees	9.30*** [6.89,11.71]	8.63*** [6.41,10.86]	4.58*** [2.57,6.60]	5.04*** [3.00,7.09]
Non-Procedural Disenrollees	0.02 [-0.93,0.98]	0.68 [-0.20,1.56]	0.56 [-0.24,1.35]	1.26*** [0.45,2.07]
By Sex				
Women (Comparison Subgroup)				
Procedural Disenrollees	2.77*** [0.70,4.83]	2.74*** [0.77,4.70]	1.13 [-0.58,2.85]	0.88 [-0.86,2.62]
Non-Procedural Disenrollees	-1.32*** [-2.19,-0.45]	-1.16*** [-1.99,-0.33]	-0.85** [-1.57,-0.12]	-1.16*** [-1.90,-0.43]
Men				

© 2025 Soni A et al. *JAMA Health Forum.*

Procedural Disenrollees	3.61*** [0.90,6.32]	3.67*** [1.19,6.15]	4.00*** [1.62,6.39]	4.50*** [2.09,6.91]
Non-Procedural Disenrollees	-0.80 [-1.96,0.36]	-1.37** [-2.44,-0.31]	-0.79 [-1.82,0.23]	-0.04 [-1.08,0.99]
By Parental Status				
Parents (Comparison Subgroup)				
Procedural Disenrollees	2.75** [0.55,4.95]	1.91* [-0.16,3.98]	1.86** [0.05,3.67]	1.64* [-0.18,3.47]
Non-Procedural Disenrollees	-0.00 [-0.97,0.96]	-0.62 [-1.53,0.30]	-0.65 [-1.45,0.14]	-0.09 [-0.89,0.72]
Childless Adults				
Procedural Disenrollees	3.79*** [1.37,6.21]	4.88*** [2.61,7.15]	3.03*** [0.91,5.14]	3.64*** [1.49,5.79]
Non-Procedural Disenrollees	-2.30***,b [-3.29,-1.31]	-1.98*** [-2.91,-1.06]	-1.04** [-1.90,-0.17]	-1.34*** [-2.22,-0.46]
By Age Group				
Age 18-34 (Comparison Subgroup)				
Procedural Disenrollees	5.95*** [2.50,9.41]	4.04** [0.82,7.25]	3.43** [0.55,6.32]	4.00*** [1.03,6.97]
Non-Procedural Disenrollees	0.21 [-1.24,1.67]	-0.04 [-1.39,1.31]	0.00 [-1.21,1.22]	-0.27 [-1.52,0.98]
Age 35-49				
Procedural Disenrollees	1.26 [-1.30,3.81]	3.12** [0.70,5.54]	3.32*** [1.18,5.46]	1.46 [-0.73,3.65]
Non-Procedural Disenrollees	-2.29*** [-3.39,-1.19]	-2.55*** [-3.59,-1.51]	-0.98** [-1.89,-0.06]	-2.03*** [-2.97,-1.09]
Age 50-64				
Procedural Disenrollees	1.71 [-1.00,4.42]	2.35* [-0.21,4.91]	-0.55 [-2.94,1.83]	0.72 [-1.60,3.05]
Non-Procedural Disenrollees	-1.74*** [-2.91,-0.56]	-1.17** [-2.28,-0.06]	-1.97*** [-3.01,-0.94]	0.35 [-0.66,1.37]

© 2025 Soni A et al. *JAMA Health Forum.*

Panel B. Functional Health

	Difficulty Seeing	Difficulty Hearing	Difficulty Remembering	Difficulty Walking/ Climbing Stairs	Difficulty Bathing/ Dressing	Difficulty Understanding
By Race and Ethnicity						
Black, non-Hispanic						
Procedural Disenrollees	-2.42[*,b] [-4.99,0.15]	-0.94[b] [-2.44,0.55]	0.34 [-2.40,3.08]	-3.43[**] [-6.53,-0.33]	-1.07 [-2.86,0.72]	-0.24 [-1.57,1.08]
Non-Procedural Disenrollees	1.41[**] [0.27,2.55]	-1.11[***,c] [-1.78,-0.45]	-0.59 [-1.81,0.63]	-3.71[***] [-5.09,-2.33]	-1.48[***] [-2.28,-0.69]	-0.67[**] [-1.25,-0.08]
Hispanic						
Procedural Disenrollees	3.64[***] [1.22,6.05]	0.62 [-0.92,2.16]	0.21 [-2.59,3.01]	-0.49 [-2.94,1.97]	1.45[*] [-0.18,3.08]	0.32 [-1.13,1.76]
Non-Procedural Disenrollees	1.16[**] [0.05,2.26]	-0.28 [-0.99,0.42]	-0.96 [-2.24,0.32]	-2.96[***] [-4.09,-1.84]	-0.70[*] [-1.44,0.05]	-0.27 [-0.93,0.39]
Other race, non-Hispanic						
Procedural Disenrollees	1.54 [-1.46,4.54]	-0.02 [-2.30,2.27]	7.39[***] [3.58,11.20]	-0.90 [-4.23,2.42]	-0.54 [-2.84,1.76]	-0.81 [-2.83,1.20]
Non-Procedural Disenrollees	-1.47[**] [-2.77,-0.18]	-0.85[*] [-1.83,0.14]	-2.58[***] [-4.22,-0.93]	-2.73[***] [-4.16,-1.29]	-0.68 [-1.68,0.31]	-1.29[***,c] [-2.16,-0.42]
White, non-Hispanic (Comparison Subgroup)						
Procedural Disenrollees	2.81[***] [1.47,4.16]	2.64[***] [1.65,3.63]	1.43 [-0.48,3.34]	-0.98 [-2.65,0.70]	0.89[*] [-0.09,1.86]	-0.65[*] [-1.42,0.12]
Non-Procedural Disenrollees	0.21 [-0.32,0.75]	-0.02 [-0.41,0.37]	-1.30[***] [-2.06,-0.55]	-1.75[***] [-2.41,-1.09]	-0.40[**] [-0.79,-0.02]	-0.12 [-0.43,0.18]
By Sex						
Women (Comparison Subgroup)						
Procedural Disenrollees	1.20[*] [-0.07,2.47]	0.32 [-0.49,1.12]	1.55[*] [-0.05,3.15]	-2.22[***] [-3.65,-0.79]	-0.04 [-0.90,0.82]	-0.42 [-1.07,0.23]
Non-Procedural Disenrollees	0.45 [-0.09,0.98]	-0.19 [-0.53,0.15]	-0.91[***] [-1.58,-0.23]	-2.65[***] [-3.26,-2.05]	-0.60[***] [-0.97,-0.24]	-0.21 [-0.48,0.07]
Men						
Procedural Disenrollees	3.18[***] [1.61,4.75]	2.23[***] [0.99,3.48]	1.04 [-1.05,3.14]	-0.31 [-2.28,1.66]	1.10[*] [-0.13,2.33]	-0.22 [-1.34,0.90]
Non-Procedural Disenrollees	0.56 [-0.12,1.23]	-0.62[**] [-1.16,-0.09]	-1.38[***] [-2.28,-0.48]	-2.82[***] [-3.66,-1.97]	-0.63[**] [-1.16,-0.11]	-0.44[*] [-0.92,0.04]

© 2025 Soni A et al. *JAMA Health Forum.*

By Parental Status						
Parents (Comparison Subgroup)						
Procedural Disenrollees	1.67**<br>[0.35,3.00]	1.16***<br>[0.28,2.03]	1.31<br>[-0.34,2.96]	-0.29<br>[-1.64,1.06]	0.99**<br>[0.15,1.83]	-0.23<br>[-0.92,0.46]
Non-Procedural Disenrollees	1.13***<br>[0.54,1.71]	-0.45**<br>[-0.84,-0.07]	-0.05<br>[-0.78,0.68]	-1.50***<br>[-2.09,-0.90]	-0.65***<br>[-1.02,-0.28]	-0.18<br>[-0.49,0.12]
Childless Adults						
Procedural Disenrollees	2.63***<br>[1.16,4.10]	1.19**<br>[0.14,2.23]	1.35<br>[-0.59,3.28]	-2.38**<br>[-4.28,-0.47]	-0.25<br>[-1.40,0.89]	-0.47<br>[-1.41,0.46]
Non-Procedural Disenrollees	-0.10<br>[-0.70,0.49]	-0.24<br>[-0.67,0.19]	-2.32***,b<br>[-3.11,-1.53]	-3.63***,a<br>[-4.41,-2.85]	-0.68***<br>[-1.15,-0.21]	-0.50**<br>[-0.88,-0.12]
By Age Group						
Age 18-34 (Comparison Subgroup)						
Procedural Disenrollees	0.76<br>[-0.96,2.49]	0.81<br>[-0.17,1.80]	3.69***<br>[0.99,6.38]	0.63<br>[-0.72,1.98]	1.78**<br>[0.35,3.21]	-0.50<br>[-1.76,0.76]
Non-Procedural Disenrollees	1.57***<br>[0.85,2.29]	-0.28<br>[-0.70,0.13]	0.02<br>[-1.11,1.16]	-1.60***<br>[-2.17,-1.04]	-0.22<br>[-0.82,0.38]	-0.62**<br>[-1.15,-0.09]
Age 35-49						
Procedural Disenrollees	0.83<br>[-0.65,2.31]	1.03**<br>[0.06,2.01]	-0.47<br>[-2.45,1.51]	-1.52*<br>[-3.17,0.13]	-0.04<br>[-1.07,0.99]	0.22<br>[-0.64,1.08]
Non-Procedural Disenrollees	-0.19<br>[-0.83,0.45]	-0.23<br>[-0.65,0.19]	-2.31***<br>[-3.16,-1.46]	-2.22***<br>[-2.93,-1.51]	-1.08***<br>[-1.52,-0.64]	-0.09<br>[-0.46,0.28]
Age 50-64						
Procedural Disenrollees	5.53***,c<br>[3.51,7.55]	1.91**<br>[0.36,3.46]	0.68<br>[-1.41,2.76]	-3.70***<br>[-6.46,-0.94]	-0.82<br>[-2.12,0.48]	-0.83*<br>[-1.77,0.11]
Non-Procedural Disenrollees	-0.09<br>[-0.96,0.79]	-0.55<br>[-1.22,0.12]	-1.19**<br>[-2.09,-0.28]	-5.75***,a<br>[-6.95,-4.56]	-0.67**<br>[-1.24,-0.11]	-0.07<br>[-0.48,0.34]

© 2025 Soni A et al. *JAMA Health Forum*.

Panel C. Financial Security

	Any Insurance	Often Not Enough to Eat
By Race and Ethnicity		
Black, non-Hispanic		
Procedural Disenrollees	-48.12[***]<br>[-49.95,-46.30]	2.70[*]<br>[-0.26,5.65]
Non-Procedural Disenrollees	-27.70[***]<br>[-28.52,-26.89]	1.02<br>[-0.29,2.34]
Hispanic		
Procedural Disenrollees	-59.50[***]<br>[-61.31,-57.70]	5.67[***]<br>[3.43,7.91]
Non-Procedural Disenrollees	-31.65[***,c]<br>[-32.48,-30.83]	0.07<br>[-0.96,1.09]
Other race, non-Hispanic		
Procedural Disenrollees	-44.23[***]<br>[-46.11,-42.34]	1.48<br>[-1.69,4.66]
Non-Procedural Disenrollees	-17.57[***,a]<br>[-18.39,-16.76]	-0.45<br>[-1.82,0.93]
White, non-Hispanic (Comparison Subgroup)		
Procedural Disenrollees	-54.32[***]<br>[-55.22,-53.41]	3.15[***]<br>[1.70,4.60]
Non-Procedural Disenrollees	-24.46[***]<br>[-24.81,-24.10]	-0.21<br>[-0.78,0.37]
By Sex		
Women (Comparison Subgroup)		
Procedural Disenrollees	-53.49[***]<br>[-54.30,-52.67]	2.75[***]<br>[1.45,4.06]
Non-Procedural Disenrollees	-26.77[***]<br>[-27.12,-26.43]	0.42<br>[-0.13,0.97]
Men		
Procedural Disenrollees	-53.58[***]<br>[-54.84,-52.31]	4.62[***]<br>[2.88,6.36]
Non-Procedural Disenrollees	-25.22[***]<br>[-25.76,-24.68]	-0.42<br>[-1.17,0.33]
By Parental Status		
Parents (Comparison Subgroup)		
Procedural Disenrollees	-55.26[***]<br>[-56.22,-54.31]	4.19[***]<br>[2.81,5.57]
Non-Procedural Disenrollees	-28.81[***]<br>[-29.23,-28.39]	0.46<br>[-0.15,1.07]
Childless Adults		
Procedural Disenrollees	-51.32[***]<br>[-52.30,-50.33]	3.14[***]<br>[1.59,4.69]
Non-Procedural Disenrollees	-23.24[***,a]<br>[-23.64,-22.84]	-0.28<br>[-0.92,0.35]

© 2025 Soni A et al. *JAMA Health Forum*.

By Age Group

Age 18-34 (Comparison Subgroup)		
Procedural Disenrollees	-55.70*** [-57.30,-54.10]	1.90** [0.05,3.74]
Non-Procedural Disenrollees	-30.11*** [-30.77,-29.45]	0.42 [-0.36,1.19]
Age 35-49		
Procedural Disenrollees	-56.12*** [-57.19,-55.05]	4.78*** [3.02,6.54]
Non-Procedural Disenrollees	-26.18***,c [-26.64,-25.72]	-0.31 [-1.06,0.45]
Age 50-64		
Procedural Disenrollees	-46.79*** [-47.76,-45.81]	4.32*** [2.47,6.17]
Non-Procedural Disenrollees	-19.72***,a [-20.14,-19.30]	0.19 [-0.62,0.99]

Notes: Authors' calculations based on Household Pulse Survey, Week 53 through Cycle 9 (which covers January 2023 to September 2024). We restricted the study sample to recent adult Medicaid enrollees (N=131,384). Table displays the regression-adjusted percentage point difference between those who disenrolled from Medicaid because they could not complete the renewal process (i.e., procedural disenrollees), those who disenrolled for some other reason (i.e., non-procedural disenrollees), and current Medicaid enrollees (reference category). Regressions control for respondents' age, sex, race and ethnicity, educational attainment, marital status, parental status, household size, household income, employment status, and state of residence. Estimates are weighted by HPS sampling weights. 95% confidence intervals are in brackets.

For heterogeneity tests by race and ethnicity, respondents' race and ethnicity are based on self reports and HPS categorization scheme. "White, non-Hispanic" includes those in the HPS category "White, Alone" and not of Hispanic, Latino, or Spanish origin. "Black, non-Hispanic" includes those in the HPS category "Black, Alone" and not of Hispanic, Latino, or Spanish origin. "Other race, non-Hispanic" includes those in the HPS categories "Asian, Alone" or "Any other race alone, or race in combination" and not of Hispanic, Latino, or Spanish origin. "Hispanic" includes those who responded "Yes, of Hispanic, Latino, or Spanish origin."

* $p < 0.10$, ** $p < 0.05$, *** $p < 0.01$

Letter subscripts indicate whether coefficient is statistically different from comparison subgroup with [a] p<0.01, [b] p < 0.05, and [c] p<0.10.

© 2025 Soni A et al. *JAMA Health Forum*.

eTable 4. Sensitivity Analyses

Panel A. Mental Health

	High Anxiety	Frequent Worrying	Little Interest in Things	High Depression
Baseline Results (for Comparison)				
Procedural Disenrollees	3.25*** [1.63,4.88]	3.30*** [1.77,4.83]	2.39*** [1.01,3.77]	2.48*** [1.08,3.88]
Non-Procedural Disenrollees	-1.00*** [-1.69,-0.31]	-1.18*** [-1.83,-0.53]	-0.75** [-1.34,-0.17]	-0.65** [-1.24,-0.06]
Alternate Definitions for Mental Health Measures				
Procedural Disenrollees	2.65*** [0.75,4.54]	4.92*** [3.08,6.76]	3.06*** [1.29,4.84]	2.85*** [1.10,4.60]
Non-Procedural Disenrollees	-1.51*** [-2.32,-0.71]	-1.70*** [-2.48,-0.91]	-1.12*** [-1.87,-0.36]	-0.65* [-1.39,0.10]
Include Wave Fixed Effects				
Procedural Disenrollees	4.33*** [2.71,5.95]	4.09*** [2.56,5.62]	3.13*** [1.75,4.51]	3.28*** [1.88,4.67]
Non-Procedural Disenrollees	0.00 [-0.70,0.70]	-0.40 [-1.06,0.26]	-0.08 [-0.68,0.52]	0.08 [-0.52,0.69]
Drop State Fixed Effects				
Procedural Disenrollees	3.64*** [2.02,5.27]	3.56*** [2.04,5.09]	2.71*** [1.33,4.09]	2.89*** [1.50,4.29]
Non-Procedural Disenrollees	-0.78** [-1.47,-0.09]	-0.98*** [-1.62,-0.33]	-0.48 [-1.06,0.10]	-0.32 [-0.91,0.27]
Interviewed Before August 2023 (Before Change in HPS Lookback Period)				
Procedural Disenrollees	3.54** [0.35,6.73]	2.72* [-0.26,5.71]	5.73*** [2.98,8.48]	1.82 [-0.92,4.56]
Non-Procedural Disenrollees	0.96 [-0.34,2.25]	-0.51 [-1.72,0.70]	0.19 [-0.93,1.30]	0.19 [-0.93,1.30]
Interviewed After August 2023 (After Change in HPS Lookback Period)				
Procedural Disenrollees	4.23*** [2.39,6.08]	4.32*** [2.57,6.06]	1.72** [0.17,3.26]	3.39*** [1.81,4.98]
Non-Procedural Disenrollees	-0.93** [-1.73,-0.13]	-0.76* [-1.51,0.00]	-0.57* [-1.25,0.10]	-0.43 [-1.12,0.26]

© 2025 Soni A et al. *JAMA Health Forum.*

Panel B. Functional Health

	Difficulty Seeing	Difficulty Hearing	Difficulty Remembering	Difficulty Walking/ Climbing Stairs	Difficulty Bathing/ Dressing	Difficulty Understanding
Baseline Results (for Comparison)						
Procedural Disenrollees	2.02*** [1.04,3.00]	1.11*** [0.43,1.78]	1.39** [0.13,2.65]	-1.31** [-2.46,-0.16]	0.51 [-0.19,1.20]	-0.34 [-0.91,0.23]
Non-Procedural Disenrollees	0.54** [0.12,0.96]	-0.37** [-0.65,-0.08]	-1.10*** [-1.64,-0.57]	-2.60*** [-3.09,-2.11]	-0.61*** [-0.90,-0.31]	-0.31** [-0.55,-0.06]
Include Wave Fixed Effects						
Procedural Disenrollees	2.30*** [1.32,3.29]	1.22*** [0.54,1.90]	1.78*** [0.52,3.04]	-1.38** [-2.53,-0.22]	0.54 [-0.16,1.24]	-0.23 [-0.80,0.34]
Non-Procedural Disenrollees	0.80*** [0.37,1.22]	-0.25 [-0.54,0.05]	-0.73*** [-1.28,-0.19]	-2.65*** [-3.15,-2.16]	-0.57*** [-0.87,-0.27]	-0.20 [-0.44,0.05]
Drop State Fixed Effects						
Procedural Disenrollees	2.28*** [1.30,3.26]	1.21*** [0.54,1.89]	1.55** [0.29,2.81]	-0.87 [-2.02,0.27]	0.61* [-0.09,1.31]	-0.22 [-0.79,0.34]
Non-Procedural Disenrollees	0.74*** [0.33,1.16]	-0.27* [-0.55,0.02]	-1.02*** [-1.55,-0.48]	-2.24*** [-2.73,-1.76]	-0.52*** [-0.81,-0.22]	-0.23* [-0.47,0.01]
Interviewed Before August 2023 (Before Change in HPS Lookback Period)						
Procedural Disenrollees	3.86*** [1.93,5.78]	1.98*** [0.64,3.32]	3.93*** [1.45,6.42]	-0.97 [-3.12,1.18]	-0.26 [-1.52,0.99]	-0.90* [-1.92,0.13]
Non-Procedural Disenrollees	1.63*** [0.85,2.41]	-0.11 [-0.65,0.44]	-0.53 [-1.54,0.48]	-3.36*** [-4.23,-2.48]	-0.57** [-1.08,-0.06]	-0.35* [-0.77,0.06]
Interviewed After August 2023 (After Change in HPS Lookback Period)						
Procedural Disenrollees	1.46** [0.34,2.58]	0.82** [0.06,1.58]	0.85 [-0.57,2.28]	-1.31* [-2.66,0.05]	0.78* [-0.06,1.63]	-0.14 [-0.83,0.55]
Non-Procedural Disenrollees	0.23 [-0.26,0.72]	-0.40** [-0.73,-0.07]	-0.93*** [-1.56,-0.31]	-2.18*** [-2.77,-1.59]	-0.67*** [-1.04,-0.30]	-0.32** [-0.62,-0.02]

© 2025 Soni A et al. *JAMA Health Forum*.

Panel C. Financial Security

	Any Insurance	Often Not Enough to Eat
Baseline Results (for Comparison)		
Procedural Disenrollees	-53.52*** [-54.20,-52.83]	3.64*** [2.60,4.67]
Non-Procedural Disenrollees	-26.10*** [-26.39,-25.81]	0.09 [-0.35,0.52]
Include Wave Fixed Effects		
Procedural Disenrollees	-53.63*** [-54.31,-52.94]	3.54*** [2.50,4.58]
Non-Procedural Disenrollees	-26.19*** [-26.49,-25.90]	-0.01 [-0.46,0.44]
Drop State Fixed Effects		
Procedural Disenrollees	-53.75*** [-54.44,-53.07]	3.84*** [2.81,4.88]
Non-Procedural Disenrollees	-26.34*** [-26.63,-26.05]	0.22 [-0.22,0.66]
Interviewed Before August 2023 (Before Change in HPS Lookback Period)		
Procedural Disenrollees	-51.93*** [-53.06,-50.81]	3.05*** [1.15,4.95]
Non-Procedural Disenrollees	-27.03*** [-27.48,-26.57]	-0.41 [-1.18,0.36]
Interviewed After August 2023 (After Change in HPS Lookback Period)		
Procedural Disenrollees	-54.09*** [-54.96,-53.22]	3.83*** [2.60,5.06]
Non-Procedural Disenrollees	-25.67*** [-26.04,-25.29]	0.29 [-0.24,0.83]

Notes: Authors' calculations based on Household Pulse Survey, Week 53 through Cycle 9 (which covers January 2023 to September 2024). We restricted the study sample to recent adult Medicaid enrollees (N=131,384). Table displays the regression-adjusted percentage point difference between those who disenrolled from Medicaid because they could not complete the renewal process (i.e., procedural disenrollees), those who disenrolled for some other reason (i.e., non-procedural disenrollees), and current Medicaid enrollees (reference category). Regressions control for respondents' age, sex, race and ethnicity, educational attainment, marital status, parental status, household size, household income, employment status, and state of residence. Estimates are weighted by HPS sampling weights. 95% confidence intervals are in brackets.

For the "Alternate Definitions of Mental Health Measures" sensitivity analysis, we classified respondents who reported feeling the mental health condition on more than half the days or nearly every day over the last 2 weeks as having the mental health condition, and those who reported feeling the condition on only several days or not at all over the last 2 weeks as not having the condition.

* $p < 0.10$, ** $p < 0.05$, *** $p < 0.01$

© 2025 Soni A et al. *JAMA Health Forum*.

eFigure 5. Scatterplot Showing the Correlation Between Household Pulse Survey's and Kaiser Family Foundation's Measures of "Procedural Disenrollment"

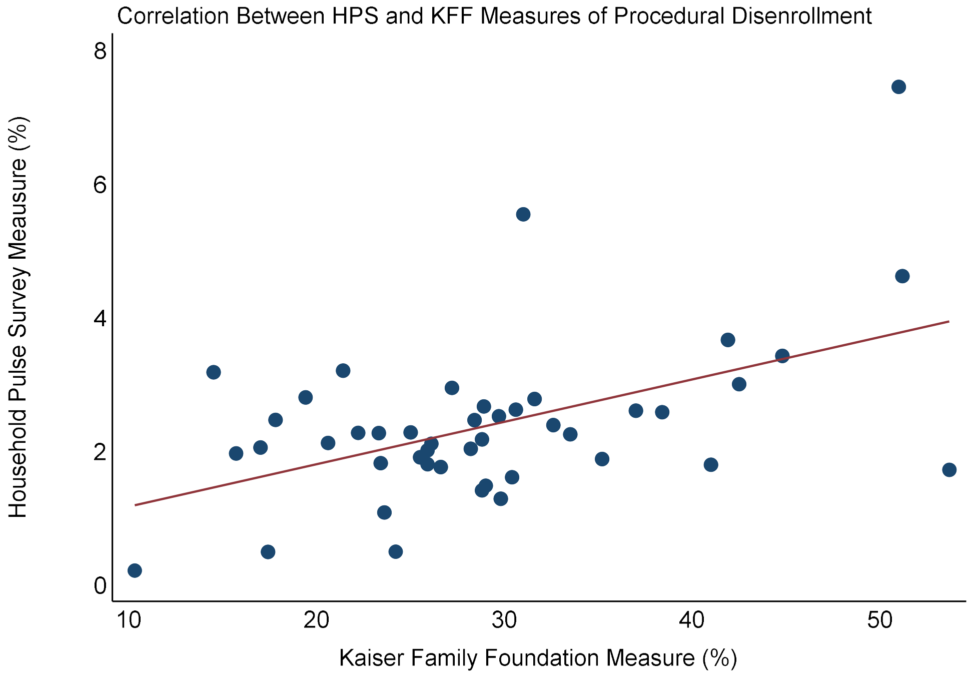

Notes: Authors' calculations based on Household Pulse Survey (HPS), Week 53 through Cycle 9 (which covers January 2023 to September 2024) and Kaiser Family Foundation (KFF)'s Medicaid Enrollment and Unwinding Tracker, Figure 3 (https://www.kff.org/report-section/medicaid-enrollment-and-unwinding-tracker-unwinding-data-archived/), which includes data as of September 12, 2024. To calculate the HPS measure, we restricted the study sample to recent adult Medicaid enrollees and calculated for each state the number of people who disenrolled from Medicaid because they could not complete the renewal process as a percentage of all recent Medicaid enrollees; estimates account for HPS sampling weights. The KFF measure is the percentage of all people with renewals due who were terminated for procedural reasons. The figure above presents each state as a dot in the scatterplot and the line of best fit in red. It should be noted that the definitions of the two measures do not completely align. The correlation between the two measures is 0.503.

© 2025 Soni A et al. *JAMA Health Forum*.
