# Supplementary material for: Health Characteristics of Adults Unable to Complete Medicaid Renewal During the Unwinding Period
Source: JAMA Health Forum. 2025 Mar 21;6(3):e250092. doi: 10.1001/jamahealthforum.2025.0092 (PMC11929023; doi:10.1001/jamahealthforum.2025.0092)
Supplement: Supplement 2. — Data Sharing Statement [file jamahealthforum-e250092-s002.pdf]

# Data Sharing Statement

Soni. Health Characteristics of Adults Unable to Complete Medicaid Renewal During the Unwinding Period. *JAMA Health Forum*. Published March 21, 2025. doi:10.1001/jamahealthforum.2025.0092

**Data**
**Data available:** Yes
**Data types:** Data (not involving human participants)
**How to access data:** Data are available by request to the corresponding author (Aparna Soni, apsoni@iu.edu).
**When available:** With publication

**Supporting Documents**
**Document types:** Statistical/analytic code
**How to access documents:** Statistical codes are available by request to the corresponding author (Aparna Soni, apsoni@iu.edu).
**When available:** With publication

**Additional Information**
**Who can access the data:** Anyone requesting the data
**Types of analyses:** For any purpose
**Mechanisms of data availability:** Publicly available data will be made available upon request.
